# A Lyapunov type inequality for fractional operators with nonsingular Mittag-Leffler kernel

**DOI:** 10.1186/s13660-017-1400-5

**Published:** 2017-06-05

**Authors:** Thabet Abdeljawad

**Affiliations:** Department of Mathematics and General Sciences, Prince Sultan University, Riyadh, Kingdom of Saudi Arabia

**Keywords:** $ABC$ fractional derivative, $ABR$ fractional derivative, Lyapunov inequality, boundary value problem, higher order, Mittag-Leffler kernel

## Abstract

In this article, we extend fractional operators with nonsingular Mittag-Leffler kernels, a study initiated recently by Atangana and Baleanu, from order $\alpha\in[0,1]$ to higher arbitrary order and we formulate their correspondent integral operators. We prove existence and uniqueness theorems for the Caputo ($ABC$) and Riemann ($ABR$) type initial value problems by using the Banach contraction theorem. Then we prove a Lyapunov type inequality for the Riemann type fractional boundary value problems of order $2<\alpha\leq3$ in the frame of Mittag-Leffler kernels. Illustrative examples are analyzed and an application as regards the Sturm-Liouville eigenvalue problem in the sense of this fractional calculus is given as well.

## Introduction

Fractional calculus [1–5] has kept attracting the interest of many authors in the last three decades or so. Some researchers have realized that finding new fractional derivatives with different singular or nonsingular kernels is essential in order to meet the need of modeling more real-world problems in different fields of science and engineering. In [6, 7] the authors studied a new type of fractional derivatives where the kernel is of exponential type and in [8, 9] the authors studied new different and interesting fractional derivatives with Mittag-Leffler kernels. Then the authors in [10, 11] studied the discrete counterparts of those new derivatives. We devote this work to an extension of the fractional calculus with Mittag-Leffler kernels to higher order, and we prove some existence and uniqueness theorems. The extension for right fractional operators and integrals is also considered to be used later by researchers in solving higher order fractional variational problems in the frame of Mittag-Leffler kernels by means of integration by parts depending on left and right fractional operators [12–14].

As an application to our extension, we prove a Lypanouv type inequality for boundary value problems with fractional operators with Mittag-Leffler kernel and of order $2< \alpha\leq3$. The limiting case of the obtained Lypanouv inequality as *α* tends to 2 from the right will give the following well-known classical Lyapunov inequality.

### Theorem 1.1

[15] *If the boundary value problem*

$$y^{\prime\prime}(t)+q(t) y(t)=0,\quad t \in(a,b),\qquad y(a)=y(b)=0, $$

*has a nontrivial solution*, *where* *q* *is a real continuous function*, *then*

1 $$ \int_{a}^{b} \bigl\vert q(s) \bigr\vert \, ds> \frac{4}{b-a}. $$

The generalization of the above Lyapunov inequality to fractional boundary value problems have been the interest of some researchers in the last few years. For examples, we refer the reader to [16–22]. For discrete fractional counterparts of Lyapunov inequalities we refer to [23] and for the *q*-fractional types we refer to [24].

The manuscript is organized as follows. In Section 2, we present some basic and necessary concepts of fractional operators with nonsingular Mittag-Leffler kernels as discussed in [8, 9, 11]. In Section 3, we extend fractional operators with nonsingular Mittag-Leffler functions and their correspondent fractional integrals to arbitrary order $\alpha>1$. In Section 4, we prove, using the Banach fixed point theorem, some existence and uniqueness theorems for Riemann ($ABR$) and Caputo ($ABC$) type initial value problems in the frame of fractional operators with Mittag-Leffler kernels, supported by some examples. In Section 5, We prove the Lyapunov type inequality for $ABR$ boundary value problems and give an example of a Sturm-Liouville eigenvalue problem. Finally, we finish by some conclusions in Section 6.

## Preliminaries

### Definition 2.1

[1]

For $\alpha>0$, $a \in\mathbb{R}$ and *f* a real-valued function defined on $[a,\infty)$, the left Riemann-Liouville fractional integral is defined by

$$\bigl({}_{a}I^{\alpha}f\bigr) (t)=\frac{1}{\Gamma(\alpha)} \int_{a}^{t} (t-s)^{\alpha-1} f(s)\,ds. $$

The right fractional integral ending at *b* is defined by

$$\bigl(I_{b}^{\alpha}f\bigr) (t)=\frac{1}{\Gamma(\alpha)} \int_{t}^{b} (s-t)^{\alpha-1} f(s)\,ds. $$

### Definition 2.2

[8, 9]

Let $f \in H^{1}(a,b)$, $a< b$, $\alpha\in[0,1]$, then the definition of the new (left Caputo) fractional derivative in the sense of Abdon and Baleanu becomes

2 $$ \bigl( {}^{ABC}_{a}D^{\alpha}f\bigr) (t)=\frac{B(\alpha)}{1-\alpha} \int_{a}^{t} f^{\prime}(x)E_{\alpha}\biggl(-\alpha\frac{(t-x)^{\alpha}}{1-\alpha} \biggr)\,dx $$

and in the left Riemann-Liouville sense has the following form:

3 $$ \bigl( {}^{ABR}_{a}D^{\alpha}f\bigr) (t)=\frac{B(\alpha)}{1-\alpha}\frac{d}{dt} \int_{a}^{t} f(x)E_{\alpha}\biggl(-\alpha \frac{(t-x)^{\alpha}}{1-\alpha} \biggr)\,dx. $$

The associated fractional integral by

4 $$ \bigl({}^{AB}_{a}I^{\alpha}f\bigr) (t)= \frac{1-\alpha}{B(\alpha)}f(t)+\frac{\alpha }{B(\alpha)}\bigl( {}_{a}I^{\alpha}f \bigr) (t). $$

Here $B(\alpha)>0$ is a normalization function satisfying $B(0)=B(1)=1$. In the right case we have

5 $$ \bigl( {}^{ABC}D_{b}^{\alpha}f\bigr) (t)= \frac{-B(\alpha)}{1-\alpha} \int_{t}^{b} f^{\prime}(x)E_{\alpha}\biggl(-\alpha\frac{(x-t)^{\alpha}}{1-\alpha} \biggr)\,dx $$

and in the right Riemann-Liouville sense it has the following form:

6 $$ \bigl( {}^{ABR}D_{b}^{\alpha}f\bigr) (t)=\frac{B(\alpha)}{1-\alpha}\frac{-d}{dt} \int _{t}^{b} f(x)E_{\alpha}\biggl(-\alpha \frac{(x-t)^{\alpha}}{1-\alpha} \biggr)\,dx. $$

The associated fractional integral by

7 $$ \bigl({}^{AB}I_{b}^{\alpha}f\bigr) (t)= \frac{1-\alpha}{B(\alpha)}f(t)+\frac{\alpha }{B(\alpha)}\bigl(I_{b}^{\alpha}f \bigr) (t). $$

In [8], it was verified that $({}^{AB}_{a}I^{\alpha}{}^{ABR}_{a}D^{\alpha}f)(t)=f(t)$ and $({}^{ABR}_{a}D^{\alpha}{}^{AB}_{a}I^{\alpha}f)(t)=f(t)$. In the right case, it was verified in [9] that $({}^{AB}I_{b}^{\alpha}{}^{ABR}D_{b}^{\alpha}f)(t)=f(t)$ and $({}^{ABR}D_{b}^{\alpha}{}^{AB}I_{b}^{\alpha}f)(t)=f(t)$. From [8] or [9] we recall the relation between the Riemann-Liouville and Caputo new derivatives:

8 $$ \bigl({}^{ABC} _{a}D^{\alpha}f\bigr) (t)=\bigl({}^{ABR} _{a}D^{\alpha}f\bigr) (t)- \frac {B(\alpha)}{1-\alpha} f(a)E_{\alpha}\biggl(-\frac{\alpha}{1-\alpha }(t-a)^{\alpha}\biggr). $$

In the next section, we extend Definition 2.2 to arbitrary $\alpha>0$.

### Lemma 2.1

[11] *For* $0< \alpha< 1$, *we have*

$$ \bigl( {}^{AB}_{a}I^{\alpha}{}^{ABC}_{a}D^{\alpha}f\bigr) (x)= f(x)-f(a) $$

*and*

$$ \bigl( {}^{AB}I_{b}^{\alpha}{}^{ABC}D_{b}^{\alpha}f\bigr) (x)= f(x)-f(b). $$

## The higher order fractional derivatives and integrals

### Definition 3.1

Let $n<\alpha\leq n+1$ and *f* be such that $f^{(n)} \in H^{1}(a,b)$. Set $\beta=\alpha-n$. Then $\beta\in(0,1]$ and we define

9 $$ \bigl( {}^{ABC}_{a}\mathbf{D}^{\alpha}f \bigr) (t)=\bigl({}^{ABC}_{a}D^{\beta}f^{(n)} \bigr) (t) $$

and in the left Riemann-Liouville sense it has the following form:

10 $$ \bigl( {}^{ABR}_{a}\mathbf{D}^{\alpha}f \bigr) (t)=\bigl({}^{ABR}_{a}D^{\beta}f^{(n)} \bigr) (t). $$

We have the associated fractional integral

11 $$ \bigl({}^{AB}_{a}\mathbf{I}^{\alpha}f \bigr) (t)= \bigl( {}_{a}I^{n}{}^{AB}_{a}I^{\beta}f\bigr) (t). $$

Note that if we use the convention that $( {}_{a}I^{0} f)(t)=f(t)$ then for the case $0<\alpha\leq1$ we have $\beta=\alpha$ and hence $( {}_{a}\mathbf {I}^{\alpha}f)(t)=( {}_{a}I^{\alpha}f)(t)$. Also, the convention $f^{(0)}(t)=f(t)$ leads to $({}^{ABR}_{a}\mathbf{D}^{\alpha}f)(t)= ({}^{ABR}_{a}D^{\alpha}f)(t)$ and $({}^{ABC}_{a}\mathbf{D}^{\alpha}f)(t)= ({}^{ABC}_{a}D^{\alpha}f)(t)$ for $0<\alpha\leq1$.

### Remark 3.1

In Definition 3.1, if we let $\alpha=n+1$ then $\beta=1$ and hence $( {}^{ABR}_{a}\mathbf{D}^{\alpha}f)(t)=({}^{ABR}_{a}\mathbf{D}^{1} f^{(n)})(t)= f^{(n+1)}(t)$. Also, by noting that $({}^{AB}_{a}I^{1}f)(t)=( {}_{a}I^{1} f)(t)$, we see that for $\alpha=n+1$ we have $({}^{AB}_{a}\mathbf {I}^{\alpha}f)(t)=( {}_{a}I^{n+1} f)(t)$. Also, for $0<\alpha\leq1$ we reobtain the concepts defined in Definition 2.2. Therefore, our generalization to the higher order case is valid.

Analogously, in the right case we have the following extension.

### Definition 3.2

Let $n<\alpha\leq n+1$ and *f* be such that $f^{(n)} \in H^{1}(a,b)$. Set $\beta=\alpha-n$. Then $\beta\in(0,1]$ and we define

12 $$ \bigl( {}^{ABC}\mathbf{D}_{b}^{\alpha}f \bigr) (t)=\bigl({}^{ABC}D_{b}^{\beta}(-1)^{n} f^{(n)}\bigr) (t), $$

and in the right Riemann-Liouville sense it has the following form:

13 $$ \bigl( {}^{ABR}\mathbf{D}_{b}^{\alpha}f \bigr) (t)=\bigl({}^{ABR}D_{b}^{\beta}(-1)^{n}f^{(n)} \bigr) (t). $$

We have the associated fractional integral

14 $$ \bigl({}^{AB}\mathbf{I}_{b}^{\alpha}f \bigr) (t)= \bigl(I_{b}^{n}{}^{AB}I_{b}^{\beta}f\bigr) (t). $$

The next proposition explains the action of the higher order integral operator ${}^{AB}_{a}\mathbf{I}^{\alpha}$ on the higher order $ABR$ and $ABC$ derivatives and, *vice versa*, the action of the $ABR$ derivative on the *AB* integral.

### Proposition 3.1

*For* $u(t)$ *defined on* $[a,b]$ *and* $\alpha\in(n,n+1]$, *for some* $n \in\mathbb{N}_{0}$, *we have*:

- $( {}^{ABR}_{a}\mathbf{D}^{\alpha}{}^{AB}_{a}\mathbf{I}^{\alpha}u)(t)=u(t)$.
- $({}^{AB}_{a}\mathbf{I}^{\alpha}{}^{ABR}_{a}\mathbf{D}^{\alpha}u)(t)=u(t)-\sum_{k=0}^{n-1} \frac{u^{(k)}(a)}{k!} (t-a)^{k}$.
- $({}^{AB}_{a}\mathbf{I}^{\alpha}{}^{ABC}_{a}\mathbf{D}^{\alpha}u)(t)=u(t)-\sum_{k=0}^{n} \frac{u^{(k)}(a)}{k!} (t-a)^{k}$.

### Proof

• By Definition 3.1 and the statement after Definition 2.2 we have

15 $$\begin{aligned} \bigl({}^{ABR}_{a}\mathbf{D}^{\alpha}{}^{AB}_{a} \mathbf{I}^{\alpha}u\bigr) (t) =& \biggl({}^{ABR}_{a}D^{\beta}\frac{d^{n}}{dt^{n}} {}_{a}I^{n} {}^{AB}_{a}I^{\beta}u\biggr) (t) \\ =&\bigl({}^{ABR}_{a}D^{\beta}{}^{AB}_{a}I^{\beta}u\bigr) (t)=u(t), \end{aligned}$$

where $\beta=\alpha-n$.

• By Definition 3.1 and the statement after Definition 2.2 we have

16 $$\begin{aligned} \bigl({}^{AB}_{a}\mathbf{I}^{\alpha}{}^{ABR}_{a} \mathbf{D}^{\alpha}u\bigr) (t) =& \bigl( {}_{a}I^{n} {}^{AB}_{a}I^{\beta}{}^{ABR}_{a}D^{\beta}u^{(n)}\bigr) (t) \\ =& {}_{a}I^{n} u^{(n)}(t)= u(t)-\sum _{k=0}^{n-1} \frac{u^{(k)}(a)}{k!} (t-a)^{k}. \end{aligned}$$

• By Lemma 2.1 applied to $f(t)=u^{(n)}(t)$ we have

17 $$\begin{aligned} \begin{aligned}[b] \bigl({}^{AB}_{a}\mathbf{I}^{\alpha}{}^{ABC}_{a} \mathbf{D}^{\alpha}u\bigr) (t) &= {}_{a}I^{n} {}_{a}I^{\beta}{}^{ABC}_{a}D^{\beta}u^{(n)}(t) \\ &= {}_{a}I^{n} \bigl[ u^{(n)}(t)- u^{(n)}(a) \bigr] \\ &=u(t)-\sum_{k=0}^{n-1} \frac{u^{(k)}(a)}{k!} (t-a)^{k}- u^{(n)}(a)\frac{ (t-a)^{n}}{n!} \\ &= u(t)-\sum_{k=0}^{n} \frac{u^{(k)}(a)}{k!} (t-a)^{k}. \end{aligned} \end{aligned}$$

 □

Similarly, for the right case we have the following.

### Proposition 3.2

*For* $u(t)$ *defined on* $[a,b]$ *and* $\alpha\in(n,n+1]$, *for some* $n \in\mathbb{N}_{0}$, *we have*:

- $( {}^{ABR}\mathbf{D}_{b}^{\alpha}{}^{AB}\mathbf{I}_{b}^{\alpha}u)(t)=u(t)$.
- $({}^{AB}\mathbf{I}_{b}^{\alpha}{}^{ABR}\mathbf{D}_{b}^{\alpha}u)(t)=u(t)-\sum_{k=0}^{n-1} \frac{(-1)^{k}u^{(k)}(b)}{k!} (b-t)^{k}$.
- $({}^{AB}\mathbf{I}_{b}^{\alpha}{}^{ABC}\mathbf{D}_{b}^{\alpha}u)(t)=u(t)-\sum_{k=0}^{n} \frac{(-1)^{k}u^{(k)}(b)}{k!} (b-t)^{k}$.

### Example 3.3

Consider the initial value problem:

18 $$ \bigl({}^{ABC}_{0}\mathbf{D}^{\alpha}y \bigr) (t)=K(t),\quad t \in[0,b], $$

where $K(t)$ is continuous on $[0,b]$. We consider two cases depending on the order *α*:

- Assume $0<\alpha\leq1$, $y(0)=c$ and $K(0)=0$. By applying ${}^{AB}_{0}\mathbf{I}^{\alpha}$ and making use of Proposition 3.1, we get the solution
  $$y(t)=c+\frac{1-\alpha}{B(\alpha)}K(t)+\frac{\alpha}{B(\alpha)}\bigl( {}_{0}I^{\alpha}K(\cdot)\bigr) (t). $$
  Notice that the condition $K(0)=0$ verifies that the initial condition $y(0)=c$. Also notice that when $\alpha\rightarrow1$ we reobtain the solution of the ordinary initial value problem $y^{\prime}(t)=K(t)$, $y(0)=c$.
- Assume $1<\alpha\leq2$, $K(0)=0 y(0)=c_{1}$, $y^{\prime}(0)=c_{2}$: By applying ${}^{AB}_{0}\mathbf{I}^{\alpha}$ and making use of Proposition 3.1 and Definition 3.1 with $\beta=\alpha -1$, we get the solution
  $$y(t)=c_{1}+c_{2}t+\frac{2-\alpha}{B(\alpha-1)} \int_{0}^{t}K(s)\,ds+\frac{\alpha -1}{B(\alpha-1)\Gamma(\alpha)} \int_{0}^{t} (t-s)^{\alpha-1}K(s)\,ds. $$
  Notice that the solution $y(t)$ verifies $y(0)=c_{1}$ without the use of $K(0)=0$. However, it verifies $y^{\prime}(0)=c_{2}$ under the assumption $K(0)=0$. Also, note that when $\alpha\rightarrow2$ we reobtain the solution of the second order ordinary initial value problem $y^{\prime \prime}(t)=K(t)$.

Next section, we prove existence and uniqueness theorems for some types of $ABC$ and $ABR$ initial value problems.

### Example 3.4

Consider the $ABC$ boundary value problem

19 $$ \bigl({}^{ABC}_{a}\mathbf{D}^{\alpha}y \bigr) (t)+q(t)y(t)=0,\quad 1< \alpha\leq2, a< t< b,\qquad y(a)=y(b)=0. $$

Then $\beta=\alpha-1$ and by Proposition 3.1 applying the operator ${}^{AB}_{a}\mathbf{I}^{\alpha}$ will result in the solution

$$y(t)=c_{1}+c_{2}(t-a)-\bigl({}^{AB}_{a} \mathbf{I}^{\alpha}q(\cdot)y(\cdot)\bigr) (t). $$

But $({}^{AB}_{a}\mathbf{I}^{\alpha}q(\cdot)y(\cdot))(t)=\frac{1-\beta}{B(\beta )} \int_{a}^{t} q(s)y(s)\,ds+\frac{\beta}{B(\beta)} {}_{a}I^{\beta+1}q(t)y(t)$. Hence, the solution has the form

$$y(t)=c_{1}+c_{2}(t-a)-\frac{2-\alpha}{B(\alpha-1)} \int_{a}^{t} q(s)y(s)\,ds-\frac {\alpha-1}{B(\alpha-1)} {}_{a}I^{\alpha}q(t)y(t), $$

or

$$y(t)=c_{1}+c_{2}(t-a)-\frac{2-\alpha}{B(\alpha-1)} \int_{a}^{t} q(s)y(s)\,ds-\frac {\alpha-1}{\Gamma(\alpha)B(\alpha-1)} \int_{a}^{t} (t-s)^{\alpha-1}q(s)y(s)\,ds. $$

The boundary conditions imply that $c_{1}=0$ and

$$c_{2}=\frac{2-\alpha}{(b-a)B(\alpha-1)} \int_{a}^{b} q(s)y(s)\,ds+\frac{\alpha -1}{(b-a)\Gamma(\alpha) B(\alpha-1)} \int_{a}^{b} (b-s)^{\alpha-1}q(s)y(s)\,ds. $$

Hence,

20 $$\begin{aligned} y(t) =& \frac{(2-\alpha)(t-a)}{(b-a)B(\alpha-1)} \int_{a}^{b} q(s)y(s)\,ds-\frac{(\alpha-1)(t-a)}{\Gamma(\alpha)(b-a)B(\alpha-1)} \int_{a}^{b} (b-s)^{\alpha-1}q(s)y(s)\,ds \\ &{}- \frac{2-\alpha}{B(\alpha-1)} \int_{a}^{t} q(s)y(s)\,ds-\frac{\alpha -1}{\Gamma(\alpha)B(\alpha-1)} \int_{a}^{t} (t-s)^{\alpha-1}q(s)y(s)\,ds. \end{aligned}$$

## Existence and uniqueness theorems for the initial value problem types

In this section we prove existence uniqueness theorems for $ABC$ and $ABR$ type initial value problems.

### Theorem 4.1

*Consider the system*

21 $$ \bigl({}^{ABC}_{a}D^{\alpha}y\bigr) (t)=f\bigl(t,y(t)\bigr), \quad t\in[a,b], 0< \alpha\leq1, \qquad y(a)=c, $$

*such that* $f(a,y(a))=0$, $A (\frac{1-\alpha}{B(\alpha)}+\frac {(b-a)^{\alpha}}{\Gamma(\alpha) B(\alpha)})<1$, *and* $|f(t,y_{1})-f(t,y_{2})|\leq A |y_{1}-y_{2}|$, $A>0$. *Here* $f:[a,b]\times\mathbb {R}\rightarrow\mathbb{R}$ *and* $y:[a,b]\rightarrow\mathbb{R}$. *Then the system* (21) *has a unique solution of the form*

22 $$ y(t)=c+{}^{AB}_{a}I^{\alpha} f \bigl(t,y(t)\bigr). $$

### Proof

First, with the help of Proposition 3.1, (8) and taking into account that $f(a,y(a))=0$, it is straightforward to prove that $y(t)$ satisfies the system (21) if and only if it satisfies (22).

Let $X=\{x: \max_{t \in[a,b]}|x(t)|<\infty\}$ be the Banach space endowed with the norm $\|x\|=\max_{t \in[a,b]}|x(t)|$. On *X* define the linear operator

$$(Tx) (t)=c+{}^{AB}_{a}I^{\alpha}f\bigl(t,x(t)\bigr). $$

Then, for arbitrary $x_{1}, x_{2} \in X$ and $t \in[a,b]$, we have by assumption

23 $$\begin{aligned} \begin{aligned}[b] \bigl\vert (Tx_{1}) (t)-(Tx_{2}) (t) \bigr\vert &= \bigl\vert {}^{AB}_{a}I^{\alpha}\bigl[ f \bigl(t,x_{1}(t)\bigr)-f\bigl(t,x_{2}(t)\bigr)\bigr] \bigr\vert \\ &\leq A \biggl(\frac{1-\alpha}{B(\alpha)}+\frac{(b-a)^{\alpha}}{\Gamma(\alpha ) B(\alpha)}\biggr) \|x_{1}-x_{2} \|, \end{aligned} \end{aligned}$$

and hence *T* is a contraction. By the Banach contraction principle, there exists a unique $x \in X$ such that $Tx=x$ and hence the proof is complete. □

### Theorem 4.2

*Consider the system*

24 $$ \bigl({}^{ABR}_{a}D^{\alpha}y\bigr) (t)=f\bigl(t,y(t)\bigr), \quad t\in[a,b], 1< \alpha\leq2,\qquad y(a)=c, $$

*such that* $\frac{A}{B(\alpha-1)} ((2-\alpha)(b-a)+\frac{(\alpha -1)(b-a)^{\alpha}}{\Gamma(\alpha+1) } )<1$, *and* $|f(t,y_{1})-f(t,y_{2})|\leq A |y_{1}-y_{2}|$, $A>0$. *Here* $f:[a,b]\times\mathbb {R}\rightarrow\mathbb{R}$ *and* $y:[a,b]\rightarrow\mathbb{R}$. *Then the system* (24) *has a unique solution of the form*

25 $$\begin{aligned} y(t) =& c+{}^{AB}_{a}\mathbf{I}^{\alpha} f\bigl(t,y(t)\bigr) \\ =& c+\frac{2-\alpha}{B(\alpha-1)} \int_{a}^{t} f\bigl(s,y(s)\bigr)\,ds+ \frac{\alpha -1}{B(\alpha-1)}\bigl( {}_{a}I^{\alpha}f\bigl(\cdot,y(\cdot) \bigr)\bigr) (t). \end{aligned}$$

### Proof

If we apply ${}^{AB}_{a}\mathbf{I}^{\alpha}$ to system (24) and make use of Proposition 3.1 with $\beta=\alpha-1$ then we obtain the representation (25). Conversely, if we apply ${}^{ABR}_{a}\mathbf{D}^{\alpha}$, make use of Proposition 3.1 and note that

$${}^{ABR}_{a}\mathbf{D}^{\alpha}={}^{ABR}_{a}D^{\beta} \frac{d}{dt}c=0, $$

we obtain the system (24). Hence, $y(t)$ satisfies the system (24) if and only if it satisfies (25).

Let $X=\{x: \max_{t \in[a,b]}|x(t)|<\infty\}$ be the Banach space endowed with the norm $\|x\|=\max_{t \in[a,b]}|x(t)|$. On *X* define the linear operator

$$(Tx) (t)=c+{}^{AB}_{a}\mathbf{I}^{\alpha}f \bigl(t,x(t)\bigr). $$

Then, for arbitrary $x_{1}, x_{2} \in X$ and $t \in[a,b]$, we have by assumption

26 $$\begin{aligned} \bigl\vert (Tx_{1}) (t)-(Tx_{2}) (t) \bigr\vert =& \bigl\vert {}^{AB}_{a}\mathbf{I}^{\alpha}\bigl[ f \bigl(t,x_{1}(t)\bigr)-f\bigl(t,x_{2}(t)\bigr)\bigr] \bigr\vert \\ \leq&\frac{A}{B(\alpha-1)} \biggl((2-\alpha) (b-a)+\frac{(\alpha -1)(b-a)^{\alpha}}{\Gamma(\alpha+1) } \biggr) \|x_{1}-x_{2}\|, \end{aligned}$$

and hence *T* is a contraction. By the Banach contraction principle, there exists a unique $x \in X$ such that $Tx=x$ and hence the proof is complete. □

## The Lyapunov inequality for the ABR boundary value problem

In this section, we prove a Lyapunov inequality for an $ABR$ boundary value problem of order $2\leq\alpha< 3$.

Consider the boundary value problem

27 $$ \bigl({}^{ABR}_{a}\mathbf{D}^{\alpha}y \bigr) (t)+q(t)y(t)=0, \quad 2\leq\alpha< 3, t \in(a,b),\qquad y(a)=y(b)=0. $$

### Lemma 5.1

$y(t)$ *is a solution of the boundary value problem* (27) *if and only if it satisfies the integral equation*

28 $$ y(t)= \int_{a}^{b} G(t,s) R\bigl(s,y(s)\bigr) \,ds, $$

*where*

$$G(t,s)= \left \{ \textstyle\begin{array}{cc} \frac{(t-a) (b-s)}{b-a} & a \leq t \leq s\leq b, \\ ( \frac{(t-a) (b-s)}{b-a}-(t-s)) & a \leq s \leq t\leq b \end{array}\displaystyle \right \} $$

*and*

$$R\bigl(t,y(t)\bigr)=({}^{AB}_{a}\mathbf{I}^{\beta}\bigl(q(\cdot)y(\cdot)\bigr) (t)=\frac{1-\beta }{B(\beta)}q(t)y(t)+\frac{\beta}{B(\beta)} \bigl( {}_{a}I^{\beta}q(\cdot)y(\cdot)\bigr) (t),\quad \beta= \alpha-2. $$

### Proof

Apply the integral ${}^{AB}_{a}\mathbf{I}^{\alpha}$ to (27) and make use of Definition 3.1 and Proposition 3.1 with $n=2$ and $\beta=\alpha-2$ to obtain

29 $$\begin{aligned} y(t) =& c_{1}+c_{2} (t-a)-\bigl( {}_{a}I^{2} R\bigl(\cdot,y(\cdot)\bigr)\bigr) (t) \\ =& c_{1}+c_{2} (t-a)- \int_{a}^{t} (t-s)R\bigl(s,y(s)\bigr)\,ds. \end{aligned}$$

The condition $y(a)=0$ implies that $c_{1}=0$ and the condition $y(b)=0$ implies that $c_{2}=\frac{1}{b-a} \int_{a}^{b} (b-s)R(s,y(s)) \,ds$ and hence

$$y(t)=\frac{t-a}{b-a} \int_{a}^{b} (b-s)R\bigl(s,y(s)\bigr) \,ds- \int_{a}^{t} (t-s)q(s) R\bigl(s,y(s)\bigr) \,ds. $$

Then the result follows by splitting the integral

$$\int_{a}^{b} (b-s)R\bigl(s,y(s)\bigr) \,ds= \int_{a}^{t} (b-s)R\bigl(s,y(s)\bigr) \,ds+ \int_{t}^{b} (b-s)R\bigl(s,y(s)\bigr) \,ds. $$

 □

### Lemma 5.2

*The Green function* $G(t,s)$ *defined in Lemma* 5.1 *has the following properties*:

- $G(t,s)\geq0$ *for all* $a\leq t,s \leq b$.
- $\max_{t \in[a,b]} G(t,s)=G(s,s)$ *for* $s \in[a,b]$.
- $H(s,s)$ *has a unique maximum*, *given by*
  $$\max_{s \in[a,b]} G(s,s)= G\biggl(\frac{a+b}{2}, \frac{a+b}{2}\biggr)=\frac{(b-a)}{4}. $$

### Proof

• It is clear that $g_{1}(t,s)=\frac{(t-a) (b-s)}{b-a}\geq0$. Regarding the part $g_{2}(t,s)=( \frac{(t-a) (b-s)}{b-a}-(t-s))$ we see that $(t-s)=\frac{t-a}{b-a}(b- (a+\frac{(s-a)(b-a)}{(t-a)}))$ and that $a+\frac{(s-a)(b-a)}{(t-a)}\geq s$ if and only if $s\geq a$. Hence, we conclude that $g_{2}(t,s)\geq0$ as well. Hence, the proof of the first part is complete.

• Clearly, $g_{1}(t,s)$ is an increasing function in *t*. Differentiating $g_{2}$ with respect to *t* for every fixed *s* we see that $g_{2}$ is a decreasing function in *t*.

• Let $g(s)=G(s,s)=\frac{(s-a)(b-s)}{b-a}$. Then one can show that $g^{\prime}(s)=0$ if $s=\frac{a+b}{2}$ and hence the proof is concluded by verifying that $g(\frac{a+b}{2})=\frac{b-a}{4}$. □

In the next lemma, we estimate $R(t, y(t))$ for a function $y \in C[a,b]$.

### Lemma 5.3

*For* $y \in C[a,b]$ *and* $2<\alpha\leq3$, $\beta=\alpha-2$, *we have for any* $t \in[a,b]$

$$\bigl\vert R\bigl(t,y(t)\bigr) \bigr\vert \leq T(t) \|y\|, $$

*where*

$$T(t)= \biggl[ \frac{3-\alpha}{B(\alpha-2)} \bigl\vert q(t) \bigr\vert + \frac{\alpha-2}{B(\alpha -2)} \bigl( {}_{a}I^{\alpha-2} \bigl\vert q(\cdot) \bigr\vert \bigr) (t) \biggr]. $$

### Theorem 5.4

*If the boundary value problem* (27) *has a nontrivial solution*, *where* $q(t)$ *is a real*-*valued continuous function on* $[a,b]$, *then*

30 $$ \int_{a}^{b} T(s)\,ds> \frac{4}{b-a}. $$

### Proof

Assume $y \in Y=C[a,b]$ is a nontrivial solution of the boundary value problem (27), where $\|y\|=\sup_{t \in[a,b]}|y(t)|$. By Lemma 5.1, *y* must satisfy

$$y(t)= \int_{a}^{b} G(t,s) R\bigl(s,y(s)\bigr)\,ds. $$

Then, by using the properties of the Green function $G(t,s)$ proved in Lemma 5.2 and Lemma 5.3, we come to the conclusion that

$$\|y\|\leq\frac{b-a}{4} \int_{a}^{b} T(s)\,ds \|y\|. $$

From this (30) follows. □

### Remark 5.1

Note that if $\alpha\rightarrow2^{+}$, then $T(t)$ tends to $|q(t)|$ and hence one obtains the classical Lyapunov inequality (1).

### Example 5.1

Consider the following $ABR$ Sturm-Liouville eigenvalue problem (SLEP) of order $2<\alpha\leq3$:

31 $$ \bigl({}^{ABR}_{0}\mathbf{D}^{\alpha}y \bigr) (t)+\lambda y(t)=0,\quad 0< t< 1,\qquad y(0)=y(1)=0. $$

If *λ* is an eigenvalue of (31), then by Theorem 5.4 with $q(t)=\lambda$, we have

32 $$\begin{aligned} T(t) =& \biggl[ \frac{3-\alpha}{B(\alpha-2)}|\lambda|+\frac{\alpha -2}{B(\alpha-2)} {}_{0}I^{\alpha-2}|\lambda| \biggr] \\ =& |\lambda| \biggl[ \frac{3-\alpha}{B(\alpha-2)}+\frac{\alpha -2}{B(\alpha-2)} \frac{t^{\alpha-2}}{\Gamma(\alpha-1)} \biggr]. \end{aligned}$$

Hence, we must have

$$\int_{0}^{1} T(s)\,ds=|\lambda| \biggl[ \frac{3-\alpha}{B(\alpha-2)}+\frac {\alpha-2}{\Gamma(\alpha)B(\alpha-2)} {} \biggr]>4. $$

Notice that the limiting case $\alpha\rightarrow2^{+}$ implies that $|\lambda|>4$. This is the lower bound for the eigenvalues of the ordinary eigenvalue problem:

$$y^{\prime\prime}(t)+\lambda y(t)=0, \quad 0< t< 1,\qquad y(0)=y(1)=0. $$

## Conclusions

We have extended the order of the fractional operators with nonsingular Mittag-Leffler function kernels from order $\alpha\in [0,1]$ to arbitrary order $\alpha>1$. Their corresponding higher order integral operators have been defined as well and confirmed. The right fractional extension is also considered. We proved existence and uniqueness theorems by means of the Banach fixed point theorem for initial value problems in the frame of $ABC$ and $ABR$ derivatives. We realized that the condition $f(a,y(a))=0$ is necessary to guarantee a unique solution and hence the fractional linear initial value problem with constant coefficients results in the trivial solution unless the order is a positive integer. As an application to our extension, we proved a Lyapunov type inequality for a $ABR$ boundary value problem with order $2<\alpha\leq 3$ and then obtained the classical ordinary case when *α* tends to 2 from the right. This is different from the classical fractional case, where the Lyapunov inequality was proved for a fractional boundary problem of order $1<\alpha\leq2$ and the classical ordinary case was recovered when *α* tends to 2 from the left.
